# Thermoelectric Mixed Thick-/Thin Film Microgenerators Based on Constantan/Silver

**DOI:** 10.3390/ma11010115

**Published:** 2018-01-12

**Authors:** Mirosław Gierczak, Joanna Prażmowska-Czajka, Andrzej Dziedzic

**Affiliations:** Faculty of Microsystem Electronics and Photonics, Wrocław University of Science and Technology, Janiszewskiego 11/17, 50-372 Wrocław, Poland; joanna.prazmowska@pwr.edu.pl (J.P.-C.); andrzej.dziedzic@pwr.edu.pl (A.D.)

**Keywords:** thermocouple, thermoelectricity, microgenerator, constantan, silver, Seebeck coefficient

## Abstract

This paper describes the design, manufacturing and characterization of newly developed mixed thick-/thin film thermoelectric microgenerators based on magnetron sputtered constantan (copper-nickel alloy) and screen-printed silver layers. The thermoelectric microgenerator consists of sixteen thermocouples made on a 34.2 × 27.5 × 0.25 mm^3^ alumina substrate. One of thermocouple arms was made of magnetron-sputtered constantan (Cu-Ni alloy), the second was a Ag-based screen-printed film. The length of each thermocouple arm was equal to 27 mm, and their width 0.3 mm. The distance between the arms was equal to 0.3 mm. In the first step, a pattern mask with thermocouples was designed and fabricated. Then, a constantan layer was magnetron sputtered over the whole substrate, and a photolithography process was used to prepare the first thermocouple arms. The second arms were screen-printed onto the substrate using a low-temperature silver paste (Heraeus C8829A or ElectroScience Laboratories ESL 599-E). To avoid oxidation of constantan, they were fired in a belt furnace in a nitrogen atmosphere at 550/450 °C peak firing temperature. Thermoelectric and electrical measurements were performed using the self-made measuring system. Two pyrometers included into the system were used for temperature measurement of hot and cold junctions. The estimated Seebeck coefficient, *α* was from the range 35 − 41 µV/K, whereas the total internal resistances *R* were between 250 and 3200 ohms, depending on magnetron sputtering time and kind of silver ink (the resistance of a single thermocouple was between 15.5 and 200 ohms).

## 1. Introduction

Small and cost-effective thermoelectric microgenerators based on the Seebeck effect in semiconductors or metals usually convert waste thermal energy directly into useable electrical energy. They are potential energy sources for low-power autonomous microsystems. The most commonly investigated thermoelectric materials, sensors and microgenerators are based on metals, materials based on silicon and/or germanium (SiGe, silicides, germanides), Bi_2_Te_3_, materials based on elements from group V (As, Sb, Bi) and group VI (Se, Te), PGEC materials (phonon-glass, electron-crystal), e.g., skutterudites, intermetallic clathrates or half-Heusler alloys), TAGS (Te-Ag-Ge-Sb) or LAST (Pb-Sb-Ag-Te) systems, and oxides with metallic conductivity or Thermoelectric Materials Functionally Graded (TMFG) [1,2,3,4,5,6,7,8,9,10]. It should be noted that some of the previously reported highly efficient thermoelectric compositions (mostly semiconductive ones, such as half-Heuslers, lead telluride and germanium telluride) can be used for power-generation applications [11].

Various methods are used for the fabrication of modern thermoelectric microsensors (e.g., temperature [12,13], heat flux [14,15], thermal insolation [15,16,17], laser power [1,18,19], Seebeck nanoantennas for solar energy harvesting [20] or calorimeters [21]) and microgenerators [1,2,3,4,5,6,7,10,22,23,24,25,26,27]—classical semiconductor technology and silicon micromachining [1], volume micromachining [1,5,6,9,25] (where, for example, vapor phase soldering is used to improve solder joint quality and reliability of the various microgenerator parts [28]), plasma spraying and laser patterning [29,30], thin-film deposition (evaporation, magnetron sputtering, electrochemical deposition) [3,8,24,25,31], thick-film technology (planar, 3D, on flexible substrates, alumina or LTCC ones) [2,4,6,7,10,11,12,13,22,26,32,33,34,35].

In general, thick-film technology is cheaper than thin-film technology, but so far, the largest Seebeck coefficient achieved for screen-printed metallic films was equal to 24 µV/K (Ag/Ni system [4,18]). On the other hand, mixed (thick/thin) film microgenerators fabricated and investigated at the Wrocław University of Science and Technology, where one arm was made from a screen-printed Ag- or Ni-based metallic film and the second one was made from magnetron-sputtered germanium-doped films [18,36,37,38], had a larger effective Seebeck coefficient, but simultaneously exhibited a much larger internal resistance, causing a lower power output compared to thick-film metallic microgenerators.

This paper presents the design, fabrication and characterization of newly developed, cost-effective thermoelectric microgenerators. The whole thermopile is based on metallic films, realized in mixed (hybrid) technology; this means that both arms are made from metallic layers. The constantan (copper-nickel alloy) arms were fabricated using thin-film technology, whereas the silver arms were prepared using standard thick-film technology. The test structures were realized on 24 alumina (96% Al_2_O_3_) substrates with a length of 34.2 mm, width of 27.5 and thickness of 0.25 mm. Sixteen thermocouples were performed on the surface of each substrate. Fabrication of the test structures can be divided into several steps: magnetron sputtering, photolithography and etching process of the constantan layer [39], and screen-printing and firing of the Ag-based film.

The thermoelectric force as a function of the temperature difference between hot and cold junctions as well as internal resistance was measured for microgenerators differing by the thickness of the magnetron-sputtered constantan layer and the kind of silver film. Measurements of structures were performed on the self-made automatic system for characterization of thermoelectric microgenerators, consisting of two pyrometers, the heating-cooling module, computer, measuring table, DC power supply and data acquisition unit [40].

This paper also presents the results of geometrical measurements, and shows total internal resistance and generated thermoelectric force as a function of temperature difference, as well as changes of the Seebeck coefficient (*α*), internal resistance (*R*) and Power Factor (*PF*) after long-term thermal exposure (it is well known that high temperature has a negative effect on most materials). The obtained results are very interesting and promising.

## 2. Materials and Methods

### 2.1. Design

**Figure 1:**
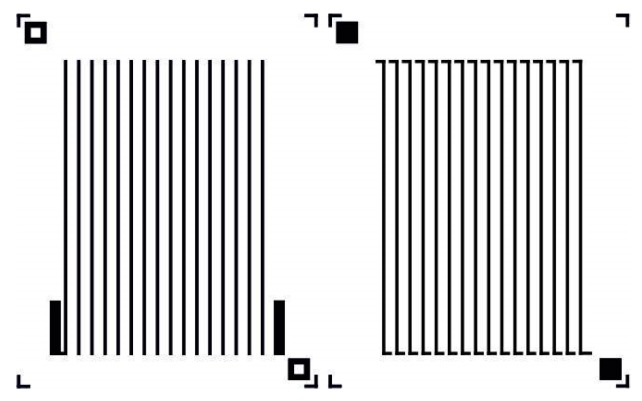
Mask pattern for microgenerators.

The design of the masks for the microgenerator is shown in Figure 1. The width of the paths and the distance between them is 300 µm, and the length is 27 mm (the length of one path is equal to 90 squares—in the future, it will be possible to reduce the planar dimensions of the microgenerator, in particular the length of its arms; this should result in a decrease in the internal resistance of the thermopile). The mask pattern for silver paths with pads is shown on the left side, whereas the mask for the constantan (copper-nickel alloy) arms is presented on the right side.

The silver mask pattern was used to create the appropriate screen, while the mask pattern for the constantan (Cu-Ni alloy) was used in the photolithography process.

### 2.2. Fabrication of Test Structures

Twenty-four alumina substrates with dimensions of 34.2 × 27.5 × 0.25 mm^3^ were chemically cleaned and degreased in the first step. Next, the constantan layer was magnetron-sputtered onto the entire substrates. The thickness of these layers was dependent on sputtering time. There were six processes, with sputtering times of 5, 10, 15, 30, 45 and 60 min, respectively.

In the next step, a photolithography process was performed for all structures to make the first thermocouple arms. The etching solution used in this process consisted of 50 mL HNO_3_, 25 mL CH_3_COOH and 25 mL of deionized water (for 100 mL solution) [39]. Depending on the thickness of the layer, the etching time varied from about 30 s (for a sputtering time of 5 min) to about 7 min (for a magnetron sputtering time of 60 min).

**Figure 2:**
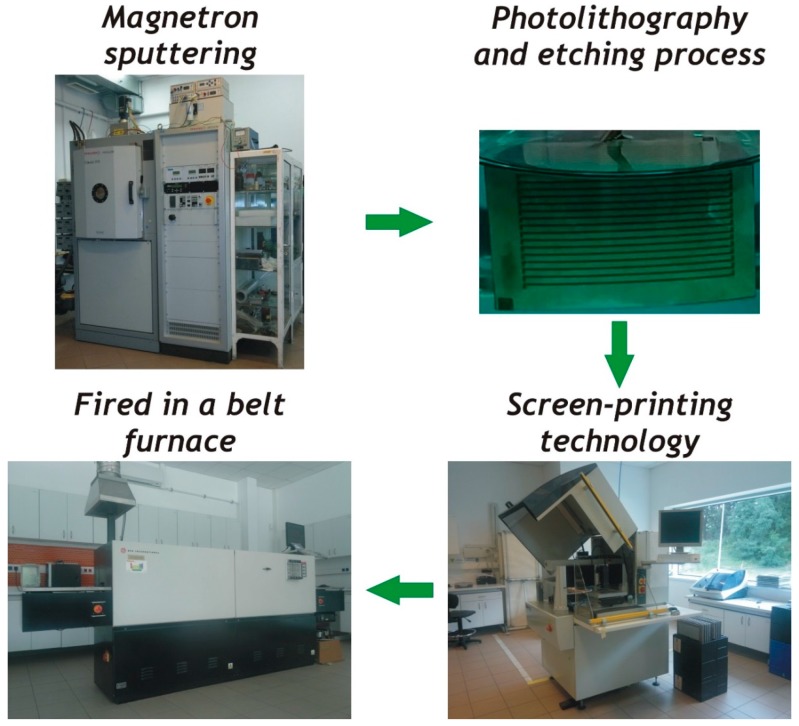
The method of fabrication of the thermoelectric microgenerator.

Then, the second arms of the thermopile were made of Ag-based ink using screen-printing technology. Low-temperature cermet silver inks—Heraeus C8829A (Heraeus, Phoenixville, PA, USA) and ESL 599-E (ESL, King of Prussia, PA, USA) were applied. These films, after printing and drying, were fired in a belt furnace in a nitrogen atmosphere for a 60 min firing cycle with 550 °C peak firing temperature for the Heraeus ink and 450 °C for the ESL one (please note that according to manufacturers’ data sheets, these inks are recommended for air firing). The method of fabrication of the thermoelectric microgenerator is shown in Figure 2. 

## 3. Results

**Figure 3:**
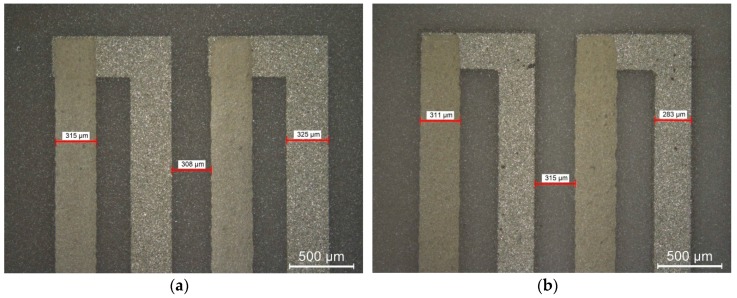
Measurement of path width (**a**) for H10.1 structure; (**b**) for H60.1 structure.

Firstly, measurements of the geometry (path width and thickness) of the constantan and silver-based layer were made. The width of the paths and the distance between them was measured with an optical microscope (Leica Microsystems, Wetzlar, Germany), whereas an optical profilometer (Taylor Hobson, Leicester, UK) was applied for thickness measurements. The results for constantan films are collected in Table 1. Moreover, the chosen microscopic pictures of the structure, together with the path widths and the distance between them, are shown in Figure 3.

It can be seen that the increase of magnetron sputtering time leads to the increase of layer thickness. This, in turn, increases the time needed to etch the correct pattern of constantan arms. This process is accompanied by undercuts, and as a result, the increase in thickness of the constantan layer leads to the width decrease of the tracks made from this material and the same the gap between the thermopile arms is increased. The width of screen-printed silver-based film is almost constant within a range of 310–315 µm, whereas its thickness *t_Ag_* is equal to about 9.5 μm. 

In the rest of this article, the structures will be marked as follows: letter H means a path made from Heraeus C8829A paste, while letter E indicates ones made from ESL 599-E composition. The number before the dot refers to the time of constantan sputtering, and the number after the dot to the sample number.

Then, the values of the total internal resistance (*R*) of the structures were measured at room temperature. The results are presented in Table 2. The resistance decreased with increasing sputtering time of the constantan layer, and was almost independent on the kind of silver film. The total internal resistance of the thermopile (*R*) is the sum of the resistance of the constantan (*R_Cu-Ni_*) and silver (*R_Ag_*) tracks, as well as the resistance of the constantan/silver junctions (*R_j_*). It can be assumed that the resistance of the silver paths and resistance of the constantan/silver junctions are constant. Moreover, it can be assumed that *R_j_* << *R_Ag_*. Thus, the systematic decrease in thermopile resistance is connected with resistance decrease of the constantan paths associated with the increase in their thickness.

Because

(1) $$R=R_{Cu-Ni}+R_{Ag}+R_{j}\approx R_{Cu-Ni}+R_{Ag}=\frac{\rho_{Cu-Ni}}{t_{Cu-Ni}}\times n_{Cu-Ni}+\frac{\rho_{Ag}}{t_{Ag}}\times n_{Ag}$$

and, according to the mask pattern shown in Figure 1, $n_{Cu-Ni}\approx 1489,$ whereas $n_{Ag}\approx 1441,$ we can estimate that $\rho_{Ag}\approx 2.85\times 10^{-8}$ Ω·m, while $\rho_{Cu-Ni}$ is from the range between $5.2\times 10^{-7}$ and $8.5\times 10^{-7}$ Ω·m.

**Figure 4:**
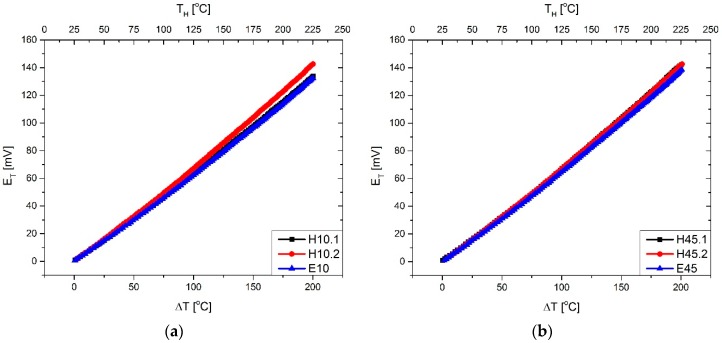
Generated thermoelectric force as a function of temperature difference (**a**) sputtering time—10 min; (**b**) sputtering time—45 min.

Thermoelectric measurements (thermoelectric force vs. temperature difference between hot and cold junction) were performed by means of a self-made measuring system [40]. Two pyrometers included in the system were used for the temperature measurement of hot and cold junctions. The chosen results are shown in Figure 4.

For each measurement, a linear regression analysis was carried out, and Seebeck coefficients were calculated for a single thermocouple. The results are collected in Table 3.

It can be seen that in the case of the thinnest (submicron) constantan film, the Seebeck coefficient was slightly lower, and that the use of Heraeus silver ink in the thermopile leads to a slightly higher value of this parameter than for film made of ESL paste.

Knowledge of Seebeck coefficient (*α*) and resistivity (*ρ*) permits the calculation of Power Factor (*PF*). In the case of thermopiles consisting of constantan and silver arms, it can be calculated from the following formula:

(2) PF=α2(ρCu−Ni+ρAg)

**Figure 5:**
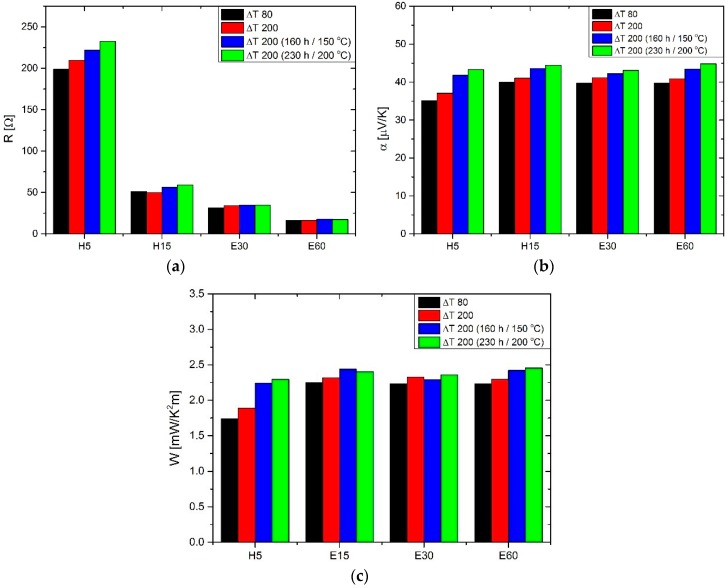
Long-term thermal exposure results (**a**) resistance; (**b**) Seebeck coefficient; (**c**) thermoelectric power factor.

Long-term thermal exposure was performed for all structures. In practically all cases, an increase in Seebeck coefficient, internal thermopile resistance and Power Factor was observed. Larger relative changes were noticed for structures with shorter magnetron sputtering time (thinner layer) of constantan. It should be noted that, despite the initial differences, the values of the Seebeck coefficient and the Power Factor after the post-process thermal aging became very close to one another. This indicates the homogenization of the internal structure of the thermopiles. Figure 5 shows the results for the selected structures. The initial measurements of the structures carried out for the temperature difference 80 °C is marked in the graphs as ΔT 80, while for a temperature difference of 200 °C—ΔT 200. The next two marks refer to the measurements after thermal exposure—first for 160 h at 150 °C and next for 230 h at 200 °C.

## 4. Discussion

This paper describes the design, fabrication and characterization of newly developed, cost-effective thermoelectric microgenerators based on magnetron-sputtered constantan (copper-nickel alloy) and screen-printed silver layers. The average value of the Seebeck coefficients calculated for the manufactured structures were in the range 35–41 µV/K—larger values were obtained for thicker films of constantan. The received value of the Seebeck coefficient is similar to the parameters of Cu-constantan or Ag-constantan wire thermocouples. After several stages of thermal aging, the Seebeck coefficient increased to a level of about 44–45 μV/K, regardless of the initial thickness of the constantan film. 

Geometrical measurements of the width of paths and the distance between them showed very similar results to the designed values.

The largest generated thermoelectric force was produced by the structure H45, and was about 143 mV at a temperature difference of around 200 °C. 

These results are very promising and the investigations are ongoing.

## Figures and Tables

**Table 1:** Geometrical properties of the constantan layers versus magnetron sputtering time.

Magnetron Sputtering Time [min]	Width of the Constantan Layer [µm]	Thickness of the Constantan Layer, *t_Cu-Ni_* [µm]	Etching Time [s]
5	327	0.4	28
10	325	0.6	33
15	327	1.0	34
30	311	2.4	159
45	315	2.8	330
60	283	3.2	420

**Table 2:** Internal resistance (at room temperature) of thermoelectric microgenerators.

Structure	Resistance [Ω]	Structure	Resistance [Ω]
H5	3180	E5	2540
H10	1310	E10	1290
H15	820	E15	1200
H30	480	E30	500
H45	365	E45	320
H60	290	E60	260

**Table 3:** Seebeck coefficient for single thermocouples.

Structure	Seebeck Coefficient, *α* [µV/K]	Structure	Seebeck Coefficient, *α* [µV/K]
H5	35.1	E5	36.9
H10	40.2	E10	37.5
H15	39.9	E15	37.2
H30	39.8	E30	39.8
H45	41.1	E45	37.9
H60	39.8	E60	39.8
